# Better characterizing sleep beliefs for personalized sleep health promotion: the French sleep beliefs scale validation study

**DOI:** 10.3389/fpubh.2023.1293045

**Published:** 2024-01-11

**Authors:** Julien Coelho, Marc Rey, Annabelle Labonne, Ana Adan, Jacques Taillard, Pierre-Alexis Geoffroy, Didier Cugy, Alexandre Dakar, Pierre Philip, Isabelle Poirot, Sylvie Royant-Parola, Sarah Hartley, Marie-Françoise Vecchierini, Jean-Arthur Micoulaud-Franchi

**Affiliations:** ^1^University Sleep Medicine Department, University Hospital of Bordeaux, Bordeaux, France; ^2^University Hospital of Bordeaux, CNRS, SANPSY, UMR 6033, Bordeaux, France; ^3^Institut National du Sommeil et de la Vigilance, Paris, France; ^4^Department of Clinical Psychology and Psychobiology, School of Psychology, University of Barcelona, Barcelona, Spain; ^5^Institute of Neurosciences, University of Barcelona, Barcelona, Spain; ^6^Département de psychiatrie et d'addictologie, AP-HP, GHU Paris Nord, DMU Neurosciences, Hopital Bichat - Claude Bernard, Paris, France; ^7^GHU Paris-Psychiatry & Neurosciences, Paris, France; ^8^Université Paris Cité, NeuroDiderot, Inserm, Paris, France; ^9^Clinique Médicale et Cardiologique d’Aressy, Aressy, France; ^10^Service de médecine physique et réadaptation pédiatrique, hospices civils de Lyon, Bron, France; ^11^Réseau Morphée, Garches, France; ^12^APHP, Hôpital Raymond Poincaré, Sleep Center, Université de Versailles Saint-Quentin en Yvelines, Garches, France; ^13^AP-HP, Hôpital Hôtel Dieu, Centre du Sommeil et de la Vigilance, Paris, France; ^14^Université Paris Descartes, Sorbonne Paris Cité, Paris, France

**Keywords:** sleep, public health, beliefs, behaviors, promotion

## Abstract

**Background:**

The Sleep Beliefs Scale (SBS) is a well-known tool to design and monitor personalized sleep health promotion at an individual and population level. The lack of an established French version limits the development of effective interventions targeting these populations. Thus, the aim of this study was to validate the French version of the SBS in a representative sample of the general population.

**Methods:**

Quota sampling was used to recruit 1,004 participants (18–65 years, mean age: 43 years, 54% of female) who underwent an online survey to complete the SBS, and to assess sleep schedules, sleep quality and disorders, and mental health. Cronbach’s α coefficient, confirmatory factor analysis, item-internal consistency (IIC), and item discriminant validity (IDV) of the SBS were computed to assess internal validity while bivariate associations with sleep schedules, sleep quality and disorders, and mental health were used to assess external convergent and discriminant validity.

**Results:**

The mean score on the SBS was 12.3 ± 4.9. Item 19 (“Quiet & Dark”) showed the highest rate of correct answers (*n* = 801, 79.8%), while item 20 (“Recovering sleep”) showed the lowest rate of correct answers (*n* = 246, 24.5%). Overall, the SBS showed satisfactory internal consistency (*α* = 0.87) and confirmed the three-factor structure proposed by the original study. All items were found consistent (IIC > 0.4) and discriminant (IIC > IDV) except for item 20 (“recovering lost sleep by sleeping for a long time”). Females, older participants, and subjects with short time-in-bed, poor sleep quality, insomnia, and circadian rhythm disorder had higher SBS scores while participants with depressive symptoms had lower SBS scores.

**Conclusion:**

We successfully translated and validated the French version of the SBS in a representative sample, making it a reliable instrument for researchers and clinicians to assess and target sleep beliefs. Correct answers vary from 25 to 80% which underlines the importance of continuing sleep health promotion campaigns by targeting poorly understood behaviors. Our findings also shed light on the fickleness of beliefs that are prone to vary within individuals across time, in step with societal changes. Several associated factors were identified, thus contributing to our understanding of sleep beliefs and offering insights for personalized approaches to enhance sleep health and overall well-being.

## 1 Introduction

Impaired sleep is one of the most prevalent health issues in the general population (1, 2) and it is thought to impact physical health, cognitive performance, emotional well-being, and overall quality of life (3). Beyond the study and the management of sleep disorders (4), the concept of sleep health has emerged as a positive framework by which individuals’ sleep may be assessed (5). Although no universal definition of sleep health exists, it has been previously defined as “*a multidimensional pattern of sleep–wakefulness, adapted to individual, social, and environmental demands, that promotes physical and mental well-being*” (5). Sleep health has gained increasing attention in recent years (6, 7) and has been linked to several physical health outcomes (e.g., cardiovascular, metabolic) (8, 9) and mental health outcomes (e.g., psychiatric, cognitive) (10, 11). This holistic approach sheds light on the importance of several sleep dimensions, including behaviors (i.e., schedules, psychotropic consumptions, and environmental factors related to sleep), beliefs (i.e., facts or ideas about sleep that are considered to be true by an individual), and attitudes (i.e., an individual’s overall feeling of like or dislike regarding a given sleep behavior) (12–17).

Sleep health promotion can be defined as the process of enabling people to increase control over, and to improve, their sleep health (18). It involves not only the diagnosis and treatment of sleep disorders but also the evaluation and modification of sleep behaviors (19, 20). Sleep-related beliefs are thought to be a major determinant of sleep behaviors and are an important target to improve sleep health (16). They are influenced by individual and societal factors (21, 22). For instance, age, gender, and circadian typology are typically associated with differences in sleep beliefs (23). Moreover, sleep beliefs may influence adherence to treatment (24), and more broadly, sleep disorders (25, 26) and the prognosis of mental disorders (27). Indeed, faulty sleep beliefs and maladaptive behaviors (e.g., about sleep duration, sleep timing, pre-sleep behavior, daytime behaviors that relate to sleep) play a role in the development of sleep disorders, particularly insomnia. Therefore, their assessment could help to develop a personalized sleep health promotion approach, defined as behavioral change interventions/management of sleep disorders tailored to individual patients or their subpopulations to achieve the highest possible therapeutic effect and to minimize side-effects (28). Repeated measures would also make it possible to measure changes in beliefs over time in line with societal changes (e.g., globalization, health disparities, changing patterns of use of technology) (29). However, to date, there is a lack of validated tools to evaluate and monitor sleep beliefs.

The Sleep Beliefs Scale (SBS) is a comprehensive instrument designed to evaluate individuals’ sleep-related beliefs (23). It measures a wide range of beliefs about factors that contribute to sleep health and was found to be an effective and reliable instrument in the original validation study (23). However, further studies are still needed to evaluate the test–retest reliability of the scale and validate it with other standard measures of sleep hygiene practice and quality, as well as in clinical samples of subjects with sleep problems such as insomnia (23). The SBS is based on an older questionnaire, the Sleep Hygiene Awareness and Practice Scale (SHAPS) (30), which was still used in its original form in some recent studies (31). This 33-item SHAPS mixes 14 items assessing sleep beliefs with 19 items evaluating sleep behaviors, while the SBS is shorter (20 items) and focuses specifically on sleep beliefs and not on sleep behaviors *per se*. It covers most of the sleep beliefs items of the SHAPS with a simplified answer format, and adds additional beliefs (e.g., thinking about one’s engagements for the next day, working intensely until late night, getting up when it is difficult to fall asleep), which can be of particular value with regard to sleep health promotion (32).

Although French is spoken by 220 million people worldwide, a validated French version of the SBS is currently unavailable. Translating questionnaires brings cultural issues into play, so before using any translated questionnaire, it is necessary to perform a transcultural validation according to specific rules and methods. Indeed, sleep behaviors and beliefs are discrepant from one community to another (33). For instance, previous studies have found that French-speaking communities are prone to sleep disturbances despite good sleep hygiene habits including a long sleep period (34). The lack of an established French version limits our understanding of sleep-related beliefs within French-speaking communities, hence hindering cross-cultural comparisons and impeding the development of effective interventions targeting these populations. Moreover, to date, the SBS has been validated only in a sample of psychology students (23). The use of a representative sample would bridge this gap and ensure the extrapolability of the psychometric validation in the whole French-speaking adult population.

The objective of this study was twofold. First, by conducting a comprehensive examination of its psychometric properties, including its reliability, factor structure, and convergent validity, we aimed to establish the French version of the SBS as a valid and reliable tool for assessing sleep-related beliefs among French-speaking individuals. Second, we explored the influence of socio-demographic characteristics, sleep schedules, sleep quality and disorders, and mental health on sleep beliefs in a representative sample of the French population. The findings will deepen our knowledge of factors influencing sleep beliefs and open new avenues for targeted interventions, ultimately advancing the field of sleep medicine toward improved health outcomes worldwide.

## 2 Materials and methods

### 2.1 Participants

Participants (18–65 years, mean age: 43 years, 54% of female) were recruited by Opinion Way, an institute specialized in conducting surveys online in a representative sample of a population (quotas sampling) *during December 2022*. Age (< 35 years, ≥ 35 years, defined based on median), sex (female, male), socio-professional status (superior, inferior, inactive), marital status (single childless, single with child, married/in couple childless, married/in couple with child), and place of residence (countryside, city <100,000 inhabitants, city >100,000 inhabitants), were noted. After receiving a detailed description of the study, participants gave their informed consent. This study was conducted in accordance with the Declaration of Helsinki.

### 2.2 Procedure

#### 2.2.1 Translation of the SBS

The translation was carried out under the auspices of the INSV (Institut National du Sommeil et de la Vigilance). The first author (Prof. A. Adan) of the original version of the SBS approved the process. A forward-backward translation was performed. The original version was translated into French independently by two French native speakers with a high level of fluency in both English and French. The back-translation into English was undertaken by two independent English native speakers and was made independently of the forward translation. The divergences observed between the back-translation and the original English version were identified and discussed. For items where cross-language agreement could not be reached, French sentences were reworded. The translated version of the SBS was administered to 10 participants and demonstrated good clarity and cultural acceptability. No further adaptations were required. The final version of the French SBS is shown in Table 1.

**Table 1:** English version/French version and frequency of response for each of item of the Sleep Beliefs Scale in a representative sample of the French population.

Item	English version	French version	Positive effect	Neither effect	Negative effect
1	Drinking alcohol in the evening	*Boire de l’alcool en soirée*	159 (15.8%)	204 (20.3%)	641 (63.8%)
2	Drinking coffee or other substances with caffeine after dinner	*Boire un café ou autre chose contenant de la caféine après le diner*	44 (4.4%)	263 (26.2%)	697 (69.4%)
3	Doing intense physical exercise before going to bed	*Faire une activité physique intense avant d’aller se coucher*	288 (28.7%)	237 (23.6%)	479 (47.7%)
4	Taking a long nap during the day	*Faire une longue sieste pendant la journée*	94 (9.4%)	245 (24.4%)	665 (66.2%)
5	Going to bed and waking up always at the same hour	*Aller au lit et se réveiller toujours à la même heure*	581 (57.9%)	293 (29.2%)	130 (13.0%)
6	Thinking about one’s engagements for the next day before falling asleep	*Penser à ce qu’il faut faire le lendemain avant de s’endormir*	100 (10.0%)	234 (23.3%)	670 (66.7%)
7	Using sleep medication regularly	*Utiliser régulièrement des médicaments pour dormir*	112 (11.2%)	207 (20.6%)	685 (68.2%)
8	Smoking before falling asleep	*Fumer avant de s’endormir*	51 (5.1%)	356 (35.5%)	597 (59.5%)
9	Diverting one’s attention and relaxing before bedtime	*Se changer les idées et se relaxer avant de l’heure du coucher*	760 (75.7%)	164 (16.3%)	80 (8.0%)
10	Going to bed 2 h later than the habitual hour	*Se coucher 2 heures plus tard que l’heure habituelle*	87 (8.7%)	322 (32.1%)	595 (59.3%)
11	Going to bed with an empty stomach	*Se coucher l’estomac vide*	86 (8.6%)	254 (25.3%)	664 (66.1%)
12	Using the bed for eating, calling on the phone, studying and other non-sleeping activities	*Utiliser le lit pour manger, téléphoner, étudier ou réaliser toute autre activité en dehors du sommeil.*	47 (4.7%)	220 (21.9%)	737 (73.4%)
13	Trying to fall asleep without having a sleep sensation	*Essayer de s’endormir sans avoir sommeil*	73 (7.4%)	320 (31.9%)	611 (60.9%)
14	Studying or working intensely until late night	*Étudier ou travailler intensément jusque tard dans la nuit*	90 (9.0%)	196 (19.5%)	718 (71.5%)
15	Getting up when it is difficult to fall asleep	*Se lever quand il est difficile de s’endormir*	261 (26.0%)	312 (31.1%)	431 (42.9%)
16	Going to bed 2 h earlier than the habitual hour	*Aller au lit 2 heures plus tôt que l’heure habituelle*	191 (19.0%)	346 (34.5%)	467 (46.5%)
17	Going to bed immediately after eating	*Aller au lit immédiatement après avoir mangé*	63 (6.3%)	202 (20.1%)	739 (73.6%)
18	Being worried about the impossibility of getting enough sleep	*Être inquiet de ne pas pouvoir dormir suffisamment*	46 (4.6%)	187 (18.6%)	771 (76.8%)
19	Sleeping in a quiet and dark room	*Dormir dans une pièce calme et obscure*	801 (79.8%)	125 (12.5%)	78 (7.8%)
20	Recovering lost sleep by sleeping for a long time	*Récupérer le manque de sommeil en dormant plus longtemps*	464 (46.2%)	294 (29.3%)	246 (24.5%)

#### 2.2.2 Assessment questionnaires

##### 2.2.2.1 Sleep beliefs scale

The SBS consists of 20 items rated by subjects according to three possible responses: neutral, positive or negative effect of the behavior described by the item on the quality and/or quantity of sleep. The SBS explores the beliefs of an individual on three factors. The first factor assesses beliefs about “sleep-incompatible behaviors,” including the influence on sleep of substance consumption (i.e., alcohol, caffeine, nicotine, sleep medication). It corresponds to eight items: 1, 2, 7, 8, 11, 12, 14, and 17. The second factor assesses beliefs about “sleep–wake cycle behaviors” including diurnal activities (i.e., physical exercise and naps). It corresponds to seven items: 3, 4, 5, 10, 16, 19, and 20. The third factor assesses beliefs about mental and physical “feelings previous to sleep” (i.e., relaxing, worries) and corresponds to 5 items: 6, 9, 13, 15, and 18. Correct answering corresponds to a negative effect on all the items except numbers 5, 9, 15 and 19, which have a positive effect. It was required that answers be related to the belief in general and not to the individual’s behavior, emphasizing the convenience of answering all the questions. The total score ranges from 0 to 20, with one point for each correct response. Thus, higher scores correspond to better beliefs.

##### 2.2.2.2 Sleep schedules

Participants were asked about their usual sleep–wake timing. They were asked what time they usually go to bed (bedtime), fall asleep (sleep latency), stay awake at night (wake after sleep onset), and get up (rise time) on workdays and on free days (35).

Time in bed was defined as the average difference between rise time and bedtime over a full week, including workdays and free days. Sleep duration was defined as the time in bed minus sleep latency and wake after sleep onset. Short time in bed and sleep duration were defined as less than the 7 h per night recommended by the National Sleep Foundation (36).

Social jetlag was defined as the difference between mid-sleep on workdays and mid-sleep on free days, mid-sleep as the median between bedtime and rise time and considered significant if at least 1 h shift (37).

Sleep timing was based on mid-sleep on free days terciles and categorized as advanced/morning timing (mid-sleep before 3:30 a.m.), neutral timing (mid-sleep between 3:30 a.m. and 4:30 a.m.) and delayed/evening timing (mid-sleep after 4:30 a.m.), as in previous studies (38).

##### 2.2.2.3 Sleep quality and disorders

Self-reported sleep quality was assessed with a single item: ‘*In general, how would you rate the quality of your sleep?*’ rated from 1: excellent, 2: very good, 3: good, 4: poor, to 5: very poor and was further categorized as good (≤ 3) or poor (≥ 4). Participants were further asked to report the current diagnosis of the following sleep disorders: insomnia disorder, circadian rhythm disorder, parasomnia, nightmares, obstructive sleep apnea syndrome and restless legs syndrome.

##### 2.2.2.4 Mental health

The Hospital Anxiety and Depression scale (HAD) was administered to assess anxiety and depressive symptoms and for external validity (39). The HAD consists of 14 items rated by a balanced four-point Likert scale. The HAD anxiety (HAD-A) consists of seven items (maximal score 21), and a score > 10 indicates clinically significant anxiety symptoms. The HAD depression (HAD-D) consists of seven items (maximal score 21), and a score > 10 indicates clinically significant depressive symptoms.

### 2.3 Statistical analyses and hypotheses

Descriptive statistics of the obtained data included frequencies and percentages of categorical variables together with means and standard deviations of continuous variables. For the validation process, we analyzed the psychometric properties of the French SBS version including internal structural validity and external validity. Data analysis was performed using R 4.1.2 (GUI 1.77 High Sierra build 8,007). For all the tests, the accepted significance level was 5%.

#### 2.3.1 Internal structural validity

Internal consistency reliability was assessed by Cronbach’s alpha coefficient. It was recalculated after items were removed and among different subgroups (participants under and above 35 years, male and female, with morning/neutral/evening timing). To confirm consistency, a coefficient of at least 0.7 was expected for each item removed.

Construct validity was assessed using confirmatory factor analysis with structural equation modeling based on the three dimensions of the SBS previously described to examine the fitness of the structure. The model fit was assessed by root mean square error of approximation (RMSEA), standardized root mean square residual (SRMR), the comparative fit index (CFI) and the Tucker-Lewis index (TLI). A RMSEA below 0.06, SRMR below 0.08, CFI higher 0.95 and TLI higher 0.95 indicate a good model fit (40).

Item-internal consistency (IIC) was assessed by correlating each item with its related dimension using Pearson’s coefficient; correlations of at least 0.4 are recommended for supporting item-internal consistency. Item discriminant validity (IDV) was assessed by determining whether items correlated better with the dimension they were hypothesized to represent compared with the other dimensions. IIC are correlations between items and the dimension that they are hypothesized to represent, and IDV are correlations between items and the other dimensions that they are not hypothesized to represent. Therefore, the IIC and IDV ranges should not largely overlap to be considered as satisfactory.

#### 2.3.2 External validity

External validity was tested by studying convergent and discriminant validity. Differences in SBS total and dimensions scores according to age, sex, sleep schedules, sleep quality and disorders, and mental health were investigated by Student’s *t*-test. As in previous studies, the rates of correct beliefs were expected to be higher among youths, women, and in subjects with morning-type chronotype (23), good sleep hygiene practice (16), and without sleep (25) and mental disorders (27).

## 3 Results

### 3.1 Sample characteristics

**Figure 1:**
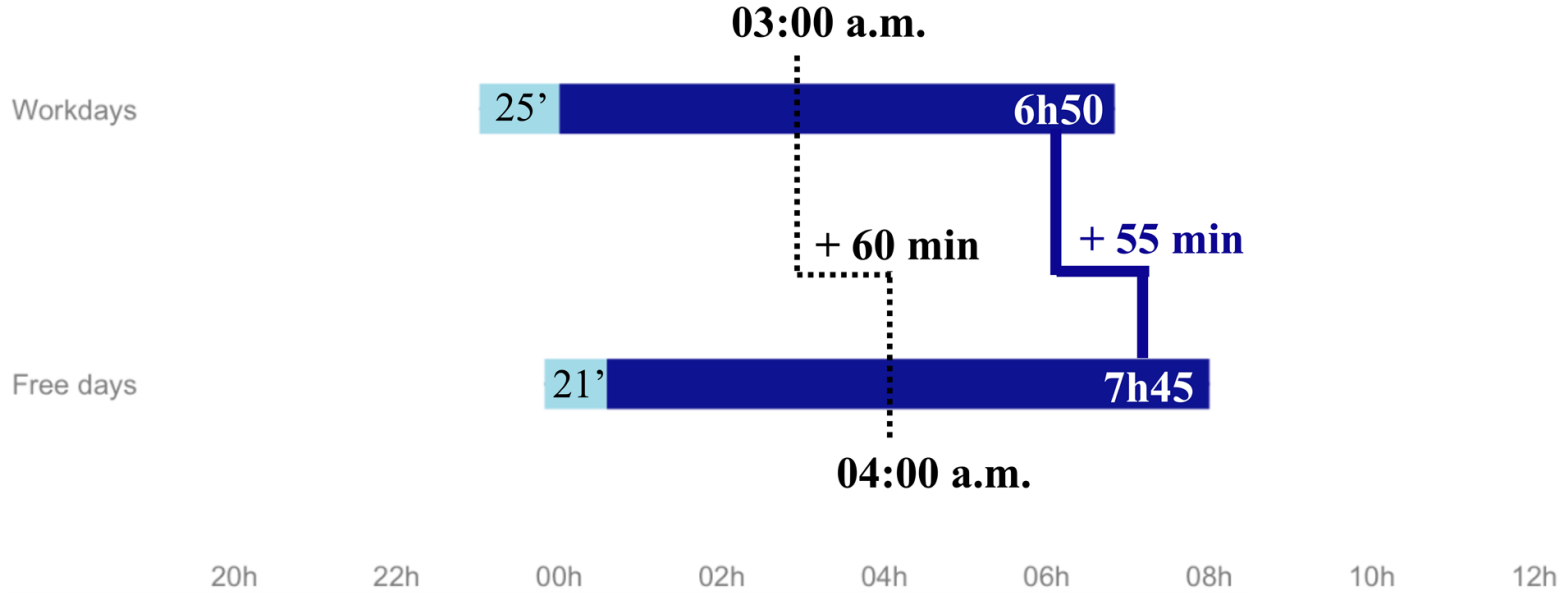
Median sleep schedules.

Evaluation was performed on 1,004 participants: mean age 43.5 years (SD = 13.4, range: [19–65]); 54.1% (543) female. Median sleep duration was 6 h50 during workdays and 7 h45 during free days and median mid-sleep was 3:00 a.m. during workdays and 4:00 a.m. during free days (Figure 1). A total of 310 (46.0%) participants reported short sleep duration (< 7 h) and 339 (33.8%) reported a significant social jetlag (≥ 1 h). In all, 316 (37.9%) participants reported a mid-sleep before 3:30 a.m. and were categorized as morning timing, 240 (28.8%) between 3:30 a.m. and 4:30 a.m., i.e., neutral timing, and 277 (33.3%) after 4:30 a.m., i.e., evening timing. A total of 370 (36.9%) participants reported poor sleep quality and 582 (58.0%) participants reported at least one sleep disorder. The most prevalent sleep disorder was insomnia disorder (*n* = 203, 20.2%), followed by circadian rhythm disorder (*n* = 174, 17.3%) and parasomnia (*n* = 174, 17.3%). Obstructive sleep apnea and restless legs syndrome were reported by 65 (6.5%) and 54 (5.4%) participants, respectively. Based on HAD scores, 312 (31.1%) patients had current clinically significant anxiety symptoms (HAD-A > 10) and 150 (14.9%) had current clinically significant depressive symptoms (HAD-D > 10). Other socio-demographical and clinical characteristics of participants are detailed in Table 1.

### 3.2 Internal validity

Results are presented in Tables 2, 3.

**Table 2:** Description of the study population.

	General population (*n* = 1,004)
Sociodemographic characteristics	
Age (continuous)	43.47 ± 13.41
< 35 years	550 (54.8%)
≥ 35 years	454 (45.2%)
Sex	
Male	461 (45.9%)
Female	543 (54.1%)
Socio-professional status	
Superior	360 (35.9%)
Inferior	396 (39.4%)
Inactive	248 (24.7%)
Marital status	
Single childless	325 (32.4%)
Single with child	102 (10.2%)
Married/In couple childless	192 (19.1%)
Married/In couple with child	385 (38.4%)
Place of residence	
Countryside	176 (17.5%)
City of less than 100,000 inhabitants	332 (33.1%)
City of more than 100,000 inhabitants	496 (49.4%)
Sleep schedules	
Time-in-bed (continuous)	7 h53 ± 93’
Short (< 7 h)	143 (18.0%)
Sleep duration (continuous) Short (< 7 h)	6 h51 ± 107’ 310 (46.0%)
Social jetlag (continuous) Significant (≥ 1 h)	1 h00 ± 63’ 339 (33.8%)
Sleep timing (continuous) Morning (mid-sleep before 3:30 a.m.) Neutral (mid-sleep between 3:30 and 4:30 a.m.) Evening (mid-sleep after 4:30 a.m.)	4:03 a.m. ± 93′ 316 (37.9%) 240 (28.8%) 277 (33.3%)
Sleep disorders	
Poor sleep quality: Yes	370 (36.9%)
Any sleep disorders: Yes	582 (58.0%)
Insomnia: Yes	203 (20.2%)
Circadian rhythm disorder: Yes	174 (17.3%)
Parasomnia: Yes	174 (17.3%)
Nightmares: Yes	92 (9.2%)
Obstructive sleep apnea syndrome: Yes	65 (6.5%)
Restless legs syndrome: Yes	54 (5.4%)
Origin of perturbation	
Occupational: Yes	348 (46.1%)
Personal: Yes	448 (44.7%)
Mental health	
Anxiety symptoms: Yes	312 (31.1%)
Depressive symptoms: Yes	150 (14.9%)

**Table 3:** Reliability (Cronbach’s *α* if one item is deleted) and validity (item-internal consistency and item-discriminant validity) for all 20 items of the scale.

Item	*α*	F1: sleep-incompatible behaviors	F2: sleep–wake cycle behaviors	F3: feelings previous to sleep	IIC	IDV
1: Alcohol	0.86	X			0.66	0.41
2: Coffee	0.86	X			0.63	0.42
3: Physical activity	0.86		X		0.56	0.39
4: Long nap	0.85		X		0.64	0.49
5: Regularity	0.86		X		0.52	0.33
6: Thinking before sleep	0.86			X	0.67	0.44
7: Sleep medication	0.86	X			0.67	0.45
8: Smoking	0.86	X			0.68	0.47
9: Relaxing	0.86			X	0.63	0.48
10: Later bedtime	0.86		X		0.59	0.48
11: Empty stomach	0.86	X			0.59	0.40
12: Non-sleeping in bed	0.85	X			0.70	0.53
13: No sleep sensation	0.86			X	0.62	0.31
14: Working late night	0.85	X			0.68	0.50
15: Getting up if no sleep	0.87			X	0.48	0.17
16: Earlier bedtime	0.86		X		0.54	0.32
17: After eating	0.86	X			0.61	0.48
18: Worrying	0.85			X	0.70	0.58
19: Quiet and dark	0.86		X		0.54	0.53
20: Recovering sleep	0.87		X		0.38	0.23

The mean score on the SBS was 12.3 (SD = 4.9; range: 0–20) in the total sample, showing a biased distribution to higher scores (correct beliefs) (Supplementary Figure S1). Item 19 (“Sleeping in a quiet and dark room”) showed the highest rate of correct answers (*n* = 801, 79.8%), followed by item 18 (“Being worried about the impossibility of getting enough sleep,” *n* = 771, 76.8%), while item 20 (“Recovering lost sleep by sleeping for a long time”) showed the lowest rate of correct answers (*n* = 246, 24.5%), followed by item 15 (“Getting up when it is difficult to fall asleep,” *n* = 261, 26.0%). The internal consistency (Cronbach’s alpha) of the SBS was satisfactory for the total sample (*α* = 0.87) and similar across age (young participants: 0.86 and old participants: 0.87), sex (male: 0.88 and female: 0.85), and sleep timing groups (morning timing: 0.88, neutral timing: 0.87 and evening timing: 0.83). Assessment of the reliability of the total scale when one item was deleted showed that they contribute significantly to the construct in all cases, with values ranging from 0.85 to 0.87. None of the items would increase Cronbach’s alpha if deleted. The three-factor structure of the SBS showed heterogeneous goodness-of-fit results in the confirmatory factor analysis (RMSEA = 0.061, SRMR = 0.050, CFI = 0.873, TLI = 0.855).

As expected, IIC were mostly higher than 0.40, except for item 20 (“Recovering lost sleep by sleeping for a long time”). The correlation of each item with its contributory dimension was higher than that with the other dimension (IIC > IDV). For “Sleep-incompatible behaviors” dimension range of IIC = 0.59–0.70 and range of IDV = 0.40–0.53. For “Sleep–wake cycle behaviors” dimension range of IIC = 0.38–0.64 and range of IDV = 0.23–0.53. For “Feelings previous to sleep” dimension range of IIC = 0.48–0.70 and range of IDV = 0.17–0.58. The items with the closest IIC and IDV were item 19 from the “Sleep–wake cycle behaviors” dimension: “Sleeping in a quiet and dark room.”

### 3.3 External validity

Results are presented in Tables 4–6.

**Table 4:** Percentage of correct responses for all 20 items of the Sleep Beliefs Scale (SBS) according to age and gender.

Item	Total sample (*n* = 1,004)	< 35 years (*n* = 550)	≥ 35 years (*n* = 454)	MW	Male (*n* = 461)	Female (*n* = 543)	MW
1: Alcohol	641 (63.8%)	337 (61.3%)	304 (67.0%)	0.062	289 (62.7%)	352 (64.8%)	0.483
2: Coffee	697 (69.4%)	388 (70.6%)	309 (68.1%)	0.396	301 (65.3%)	396 (72.9%)	**0.009**
3: Physical activity	479 (47.7%)	239 (43.5%)	240 (52.9%)	**0.003**	206 (44.7%)	273 (50.3%)	0.077
4: Long nap	665 (66.2%)	366 (66.6%)	299 (65.9%)	0.819	281 (61.0%)	384 (70.7%)	**0.001**
5: Regularity	581 (57.9%)	333 (60.6%)	248 (54.6%)	0.059	254 (55.1%)	327 (60.2%)	0.102
6: Thinking before sleep	670 (66.7%)	365 (66.4%)	305 (67.2%)	0.785	276 (59.9%)	394 (72.6%)	**<0.001**
7: Sleep medication	685 (68.2%)	371 (67.5%)	314 (69.2%)	0.563	299 (64.9%)	386 (71.1%)	**0.035**
8: Smoking	597 (59.5%)	326 (59.3%)	271 (59.7%)	0.893	272 (59.0%)	325 (59.9%)	0.785
9: Relaxing	760 (75.7%)	418 (76.0%)	342 (75.3%)	0.806	323 (70.1%)	437 (80.5%)	**<0.001**
10: Later bedtime	595 (59.3%)	346 (62.9%)	249 (54.9%)	**0.010**	264 (57.3%)	331 (61.0%)	0.236
11: Empty stomach	664 (66.1%)	361 (65.6%)	303 (66.7%)	0.713	281 (61.0%)	383 (70.5%)	**0.001**
12: Non-sleeping in bed	737 (73.4%)	383 (69.6%)	354 (78.0%)	**0.003**	331 (71.8%)	406 (74.8%)	0.289
13: No sleep sensation	611 (60.9%)	304 (55.3%)	307 (67.6%)	**<0.001**	276 (59.9%)	335 (61.7%)	0.555
14: Working late night	718 (71.5%)	399 (72.6%)	319 (70.3%)	0.426	307 (66.6%)	411 (75.7%)	**0.001**
15: Getting up if no sleep	261 (26.0%)	131 (23.8%)	130 (28.6%)	0.084	118 (25.6%)	143 (26.3%)	0.791
16: Earlier bedtime	467 (46.5%)	238 (43.3%)	229 (50.4%)	**0.024**	203 (44.0%)	264 (48.6%)	0.147
17: After eating	739 (73.6%)	388 (70.6%)	351 (77.3%)	**0.016**	317 (68.8%)	422 (77.7%)	**0.001**
18: Worrying	771 (76.8%)	409 (74.4%)	362 (79.7%)	**0.045**	339 (73.5%)	432 (79.6%)	**0.024**
19: Quiet & Dark	801 (79.8%)	429 (78.0%)	372 (81.9%)	0.122	348 (75.5%)	453 (83.4%)	**0.002**
20: Recovering sleep	246 (24.5%)	137 (24.9%)	109 (24.0%)	0.742	92 (20.0%)	154 (28.4%)	**0.002**
Total	12.34 ± 4.91	12.17 ± 4.83	12.42 ± 4.95	0.440	11.66 ± 5.18	12.91 ± 4.60	**<0.001**
F1: Sleep incompatible	5.46 ± 2.42	5.35 ± 2.48	5.50 ± 2.39	0.897	5.20 ± 2.52	5.67 ± 2.31	**0.003**
F2: Sleep–wake cycle	3.82 ± 1.78	3.86 ± 1.78	3.80 ± 1.79	0.655	3.57 ± 1.80	4.03 ± 1.74	**<0.001**
F3: Feelings	3.06 ± 1.39	2.86 ± 1.36	3.15 ± 1.40	**<0.001**	2.89 ± 1.45	3.21 ± 1.33	**<0.001**

MW, Mann–Whitney test. Bold if statistically significant (*p* < 0.05).

**Table 5:** Percentage of correct responses for all 20 items of the Sleep Beliefs Scale (SBS) in the total sample and according to sleep timing.

Item	Total sample	Morning	Neutral	Evening	KW	MW
1: Alcohol	641 (63.8%)	162 (67.5%)	218 (69.0%)	174 (62.8%)	0.136	
2: Coffee	697 (69.4%)	182 (75.8%)	214 (67.7%)	194 (70.0%)	0.166	
3: Physical activity	479 (47.7%)	133 (55.4%)	162 (51.3%)	121 (43.7%)	0.156	
4: Long nap	665 (66.2%)	163 (67.9%)	216 (68.4%)	192 (69.3%)	0.928	
5: Regularity	581 (57.9%)	156 (65.0%)	169 (53.5%)	173 (62.5%)	**0.014**	M&E > N
6: Thinking before sleep	670 (66.7%)	170 (70.8%)	207 (65.5%)	197 (71.1%)	0.079	
7: Sleep medication	685 (68.2%)	180 (75.0%)	209 (66.1%)	190 (68.6%)	0.813	
8: Smoking	597 (59.5%)	150 (62.5%)	193 (61.1%)	163 (58.8%)	0.901	
9: Relaxing	760 (75.7%)	189 (78.8%)	244 (77.2%)	219 (79.1%)	0.733	
10: Later bedtime	595 (59.3%)	159 (66.3%)	190 (60.1%)	159 (57.4%)	0.645	
11: Empty stomach	664 (66.1%)	157 (65.4%)	215 (68.0%)	185 (66.8%)	0.588	
12: Non-sleeping in bed	737 (73.4%)	192 (80.0%)	233 (73.7%)	204 (73.7%)	0.160	
13: No sleep sensation	611 (60.9%)	152 (63.3%)	195 (61.7%)	175 (63.2%)	0.905	
14: Working late night	718 (71.5%)	178 (74.2%)	225 (71.2%)	212 (76.5%)	0.335	
15: Getting up if no sleep	261 (26.0%)	59 (24.6%)	88 (27.9%)	62 (22.4%)	0.303	
16: Earlier bedtime	467 (46.5%)	113 (47.1%)	152 (48.1%)	125 (45.1%)	0.766	
17: After eating	739 (73.6%)	200 (83.3%)	227 (71.8%)	209 (75.5%)	**0.006**	M > E&N
18: Worrying	771 (76.8%)	190 (79.2%)	238 (75.3%)	229 (82.7%)	0.091	
19: Quiet and Dark	801 (79.8%)	203 (84.6%)	257 (81.3%)	227 (82.0%)	0.584	
20: Recovering sleep	246 (24.5%)	58 (24.2%)	78 (24.7%)	63 (22.7%)	0.852	
Total	12.34 ± 4.91	12.44 ± 5.06	13.11 ± 4.78	12.54 ± 4.42	0.070	
F1: Sleep incompatible	5.46 ± 2.42	5.49 ± 2.46	5.84 ± 2.31	5.53 ± 2.26	0.089	
F2: Sleep–wake cycle	3.82 ± 1.78	3.87 ± 1.82	4.10 ± 1.82	3.83 ± 1.62	0.111	
F3: Feelings	3.06 ± 1.39	3.08 ± 1.40	3.17 ± 1.35	3.18 ± 1.28	0.713	

KW, Kruskal–Wallis test; MW, Mann–Whitney test; M, morning timing; NT, neutral timing; E, evening timing. Bold if statistically significant (*p* < 0.05).

**Table 6:** Percentage of correct responses for all 20 items of the Sleep Beliefs Scale (SBS) according to sleep hygiene, sleep disorders, and mental health.

Subgroups	Total score	F1: sleep incompatible	F2: sleep–wake cycle	F3: Feelings
Time in bed				
< 7 h	11.92 ± 5.11	5.24 ± 2.57	3.77 ± 1.79	2.90 ± 1.42
≥ 7 h	12.91 ± 4.65	5.70 ± 2.28	3.99 ± 1.74	3.21 ± 1.32
value of *p*	**0.034**	ns	ns	**0.018**
Sleep duration				
< 7 h	13.13 ± 4.33	5.81 ± 2.14	4.08 ± 1.65	3.24 ± 1.27
≥ 7 h	12.73 ± 4.81	5.62 ± 2.34	3.94 ± 1.77	3.16 ± 1.36
value of p	ns	ns	ns	ns
Social jetlag				
≥ 1 h	12.70 ± 4.47	5.58 ± 2.27	3.96 ± 1.64	3.16 ± 1.30
< 1 h	12.75 ± 4.95	5.65 ± 2.40	3.95 ± 1.83	3.15 ± 1.37
value of *p*	ns	ns	ns	ns
Sleep timing				
Morning	12.44 ± 5.06	5.49 ± 2.46	3.87 ± 1.82	3.08 ± 1.40
Neutral	13.11 ± 4.78	5.84 ± 2.31	4.10 ± 1.82	3.17 ± 1.35
Evening	12.54 ± 4.42	5.53 ± 2.26	3.83 ± 1.62	3.18 ± 1.28
value of *p*	ns	ns	ns	ns
Sleep quality				
Poor	13.26 ± 4.42	5.85 ± 2.16	4.12 ± 1.71	3.29 ± 1.27
Good	11.80 ± 5.10	5.23 ± 2.52	3.64 ± 1.80	2.93 ± 1.45
value of *p*	**<0.001**	**<0.001**	**<0.001**	**<0.001**
Insomnia				
Yes	12.99 ± 4.22	5.68 ± 2.17	3.99 ± 1.59	3.32 ± 1.28
No	12.17 ± 5.06	5.40 ± 2.47	3.78 ± 1.83	3.00 ± 1.42
value of *p*	**0.019**	ns	ns	**0.002**
Circadian rhythm disorder				
Yes	13.24 ± 4.09	5.76 ± 2.16	4.20 ± 1.59	3.28 ± 1.20
No	12.15 ± 5.05	5.39 ± 2.46	3.74 ± 1.81	3.01 ± 1.43
value of *p*	**0.002**	**0.049**	**<0.001**	**0.010**
Parasomnia				
Yes	13.63 ± 3.37	6.32 ± 1.89	3.89 ± 1.05	3.42 ± 1.26
No	12.31 ± 4.93	5.44 ± 2.42	3.82 ± 1.79	3.05 ± 1.40
value of *p*	ns	ns	ns	ns
Nightmares				
Yes	12.66 ± 4.68	5.40 ± 2.52	3.95 ± 1.78	3.32 ± 1.23
No	12.30 ± 4.93	5.46 ± 2.41	3.81 ± 1.78	3.04 ± 1.41
value of *p*	ns	ns	ns	**0.042**
Obstructive sleep apnea				
Yes	12.45 ± 4.69	5.58 ± 2.47	3.83 ± 1.64	3.03 ± 1.32
No	12.33 ± 4.93	5.45 ± 2.42	3.82 ± 1.79	3.06 ± 1.40
value of *p*	ns	ns	ns	ns
Restless legs				
Yes	11.98 ± 5.12	5.17 ± 2.52	3.74 ± 1.82	3.07 ± 1.40
No	12.36 ± 4.90	5.47 ± 2.41	3.82 ± 1.78	3.06 ± 1.40
value of *p*	ns	ns	ns	ns
Anxiety symptoms				
Yes	12.56 ± 4.63	5.47 ± 2.42	3.92 ± 1.71	3.17 ± 1.28
No	12.23 ± 5.03	5.45 ± 2.42	3.77 ± 1.81	3.01 ± 1.44
value of *p*	ns	ns	ns	ns
Depressive symptoms				
Yes	11.59 ± 5.00	4.95 ± 2.55	3.75 ± 1.77	2.89 ± 1.41
No	12.47 ± 4.88	5.55 ± 2.38	3.83 ± 1.78	3.09 ± 1.39
value of *p*	**0.047**	**0.008**	ns	ns

Bold if statistically significant (*p* < 0.05).

Mean score did not differ according to age (*p* = 0.440). However, participants above 35 years showed higher rates of correct answers on the following items (3, physical activity, 12, non-sleeping in bed, 13, no sleep sensation, 16, earlier bedtime, 17: after eating, 18: worrying), while young participants acknowledged item 10 more (later bedtime). Males obtained a mean score of 11.7 (SD = 5.2) while females had a significantly higher mean score of 12.9 (SD = 3.4, *p* < 0.001) as well as the three dimensions. There were no significant associations between sleep beliefs and sleep timing except for item 5 (“Going to bed and waking up always at the same hour”) and item 17 (“Going to bed immediately after eating”), which were more acknowledged by morning timing participants (65.0 and 83.3%, respectively) and less acknowledged by neutral timing participants (53.5 and 71.8%, respectively). Regarding sleep hygiene, short time in bed (< 7 h) was associated with a lower total score (11.9 vs. 12.9, *p* = 0.034) and a lower “Feelings previous to sleep” score (2.9 vs. 3.2, *p* = 0.018). There were no differences according to sleep duration and social jetlag. Participants who reported poor sleep quality had a significantly higher total score (13.3 vs. 11.8, *p* < 0.001), a higher “Sleep incompatible behaviors” score (5.9 vs. 5.2, *p* < 0.001), a higher “Sleep–wake cycle behaviors” score (4.1 vs. 3.6, *p* < 0.001), and a higher “Feelings previous to sleep” score (3.3 vs. 2.9, *p* < 0.001). Regarding sleep disorders, participants who reported insomnia disorder had a significantly higher total score (13.0 vs. 12.2, *p* = 0.019) and a higher “Feelings previous to sleep” score (3.3 vs. 3.0, *p* = 0.002). Participants with circadian rhythm disorder had higher rates of total score and each dimension. Participants with nightmares better acknowledged items from the “Feelings previous to sleep” dimension (3.3 vs. 3.0, *p* = 0.042). Total score was lower among participants with significant depressive symptoms on the HAD (11.6 vs. 12.5, *p* = 0.047), as was the “Sleep-incompatible behaviors” score (5.0 vs. 5.6, *p* = 0.008).

## 4 Discussion

### 4.1 Key results

Our aim was to translate and validate the French version of the SBS (23), in order to make this self-rated questionnaire available for evaluating sleep beliefs in speakers of French. Moreover, this study is the first to evaluate the rate of sleep beliefs in a representative sample of the French population. The overall score on the SBS in our study (12.3) was a little lower than in the original validation article (13.1) (23). This might be due to the fact that the previous study was conducted among psychological students, a population with a high level of education and with an over-representation of females (64% vs. 54% in our study), who obtained higher SBS scores (female: 12.9 vs. male: 11.7 in our study, 13.4 vs. 12.5 in the original study) (23).

### 4.2 Internal validation: toward a better characterization of sleep beliefs

The psychometric properties of the French version were satisfactory and similar to those of the original scale (23). The internal consistency reliability was high (Cronbach’s alpha >0.87) and the item-internal consistency and the item-discriminant validity did not overlap, indicating that the French SBS has good internal homogeneity. The confirmatory factor analysis of the three-dimensional structure of the SBS showed satisfactory goodness-of-fit.

Regarding quality of discrimination, item 19 (“Quiet & Dark”) showed the lowest difference between IIC (0.54) and IDV (0.53). This lack of discrimination is probably due to a ceiling effect. Indeed, this item showed the highest rate of correct answers (79.8%), followed by item 18 (“Worrying,” 76.8% of correct answers). This is consistent with the original study which found a high rate of correct answers on these items (92.0% on item 19, 74.0% on item 18). Indeed, these two beliefs are rather common sense and it is not surprising for them to have a high response rate given their central role in sleep hygiene (41).

Regarding consistency, Item 20 (“Recovering sleep”) had the lowest IIC (0.38) followed by item 15 (“Getting up if no sleep,” IIC = 0.48). This lack of consistency is probably due to a floor effect. Indeed, these two items had the lowest rate of correct answers: 24.5 and 26.0%, respectively. This is consistent with the original study which found a low rate of correct answers on these items (46.3% on item 20, 50.8% on item 15). Nevertheless, our response rates on these items were almost 2-fold lower than in the original study, and almost half of our population answered the opposite of the expected answers by considering that “recovering lost sleep by sleeping for a long time” has a positive effect and “getting up when it is difficult to fall asleep” has a negative effect. We hypothesize that these changes may be due to the growing interest in sleep and the recent increase in prevention messages from the learned sleep societies around the world (42).

Indeed, beliefs are likely to change over time at an individual and population level (43). Among explaining factors, changes in our society are prone to modify sleep behaviors and beliefs (29). For instance, the widespread use of smartphones makes screen consumption possible in bed at night (44), while the generalization of telecommuting following the Covid-19 health crisis increased the opportunity to sleep (45). Thus, a valid and reliable instrument is needed to precisely measure changes in sleep beliefs and guide future sleep promotion campaigns to achieve greater impact by preferentially targeting the least well understood behaviors in the general population.

### 4.3 External validation: toward a personalized tool for promoting sleep health

Regarding associations with sociodemographic characteristics, females had the highest rate of correct answers in the total score and on each of the three dimensions. Older participants (≥ 35 years) had more correct answers on “Feelings previous to sleep” (3.2 vs. 2.9, *p* < 0.001).

Regarding associations with sleep schedules, we did not replicate the results of the original study on the associations between SBS scores and circadian typology (23). While participants with morning-type chronotype reported higher rates of correct answers on several items, we did not find such differences for morning or evening sleep timing (except for items 5 and 17). This discrepancy might be due to the chronotype/sleep timing assessment that was based on preferred sleep and wake time in the original study, while it was based on actual sleep timing in our study (46). However, we found that sleep beliefs vary depending on sleep schedules. Indeed, participants with a long time in bed (≥ 7 h) had higher SBS scores and “Feelings previous to sleep” scores, while there was no difference with regard to sleep duration, social jetlag or sleep timing.

Regarding associations with sleep disorders, participants who reported poor sleep quality, insomnia disorder, or circadian rhythm disorder had higher SBS scores. This surprising result is probably explained by the increased interest in the sleep of individuals with sleep disorders (25). In this specific population of patients, we hypothesize that the issue is not a lack of correct sleep beliefs, but above all the occurrence of sleep dysfunctional cognition (also called disbeliefs), with a bias producing emotional distress and heightening arousal and thus feeding the vicious cycle of poor sleep (47). These disbeliefs are even a part of the diagnostic criteria of insomnia disorder, according to the *Diagnostic and Statistical Manual of Mental Disorders* (DSM-5) and the *International Classification of Sleep Disorder* (ICSD-3) (48, 49). Of note, the Dysfunctional Beliefs and Attitudes about Sleep (DBAS) scale was specifically developed for this clinical population to guide the implementation of psychotherapy treatment for insomnia disorder (50, 51), while the SBS is more suitable for populations without sleep disorders as a tool for general sleep health promotion (23).

Regarding associations with mental health, participants with depressive symptoms had lower SBS scores as expected. Thus, the evaluation and modification of sleep beliefs in this population may be of interest. However, regarding anxiety symptoms, there was no association.

### 4.4 Limitations

First, the assessment of sleep schedules and sleep disorders was not based on objective measurement (i.e., actigraphy, polysomnography) or on validated scales. However, the use of self-reported sleep schedules is a valid and reliable tool, despite slight over-estimation of sleep duration and under-estimation of nocturnal awakenings (52). Moreover, the frequency of sleep disorders in our study is consistent with prevalence in France (15–20% of insomnia disorder, 4–6% of obstructive sleep apnea syndrome, 2–8% of restless legs syndrome) (53).

Second, our sample was limited to individuals between the ages of 18 and 65 and validation of the SBS in children and the older adults needs further study. Furthermore, the quota sampling used to perform this survey may have failed to include deprived individuals without a telephone or internet access. Future studies should evaluate sleep beliefs in these specific populations.

Third, the external validation did not include the association of sleep beliefs with sleep attitudes, as evaluated by the Sleep Practices and Attitudes Questionnaire (54) or sleep disbeliefs as evaluated by the DBAS scale (50, 51). Although consumptions are strongly related to sleep (55–57), and despite their presence in 4 of the 20 items of the SBS, they were not evaluated. These associations should be explored in further studies.

Fourth, the study was not designed to assess test–retest validation and feasibility. It would now be pertinent to assess the extent to which sleep beliefs can change and in what time frame.

### 4.5 Conclusion

We successfully translated and validated the French version of the SBS in a representative sample, making it a reliable instrument for researchers and clinicians to assess and target sleep beliefs. Correct answers vary from 25 to 80% which underlines the importance of continuing sleep health promotion campaigns by particularly targeting poorly understood behaviors. Our findings also shed light on the fickleness of beliefs that are prone to vary within individuals across time, in step with societal changes. Several associated factors were identified, thereby contributing to our understanding of sleep beliefs and offering insights for personalized approaches to enhance sleep health and overall well-being.

## Data availability statement

The raw data supporting the conclusions of this article will be made available by the authors, without undue reservation.

## Ethics statement

Ethical approval was not required for the studies involving humans because research on humans involving only anonymous questionnaires do not require ethical approval in France. The studies were conducted in accordance with the local legislation and institutional requirements. The participants provided their written informed consent to participate in this study.

## Author contributions

JC: Formal analysis, Visualization, Writing – original draft. MR: Conceptualization, Data curation, Funding acquisition, Investigation, Methodology, Project administration, Resources, Software, Supervision, Validation, Writing – review & editing. AL: Funding acquisition, Resources, Writing – review & editing. AA: Conceptualization, Methodology, Validation, Writing – review & editing. JT: Methodology, Writing – review & editing. P-AG: Methodology, Validation, Writing – review & editing. DC: Supervision, Validation, Writing – review & editing. AD: Conceptualization, Funding acquisition, Methodology, Software, Supervision, Validation, Writing – review & editing. PP: Validation, Writing – review & editing. IP: Conceptualization, Investigation, Methodology, Validation, Writing – review & editing. SR-P: Funding acquisition, Investigation, Methodology, Supervision, Validation, Writing – review & editing. SH: Funding acquisition, Investigation, Supervision, Validation, Writing – review & editing. M-FV: Conceptualization, Data curation, Funding acquisition, Investigation, Methodology, Software, Supervision, Validation, Writing – review & editing. J-AM-F: Conceptualization, Data curation, Formal analysis, Funding acquisition, Investigation, Methodology, Project administration, Resources, Software, Supervision, Validation, Visualization, Writing – original draft.
